# Joint interpretation of magnetic, transient electromagnetic, and electric resistivity tomography data for landfill characterization and contamination detection

**DOI:** 10.1038/s41598-024-83848-9

**Published:** 2024-12-23

**Authors:** Ismael M. Ibraheem, Pritam Yogeshwar, Rainer Bergers, Bülent Tezkan

**Affiliations:** Institute of Geophysics and Meteorology, University of Cologne, Pohligstrasse 3, 50969 Cologne, Germany

**Keywords:** Landfill, Contamination, Magnetics, Transient electromagnetics TEM, Electrical resistivity tomography ERT, Cologne, Germany, Geophysics, Environmental impact, Hydrogeology

## Abstract

Geophysical techniques have become increasingly crucial for characterizing landfills, offering noninvasive methods for subsurface exploration and contamination assessment. In this study, an integrated geophysical approach—utilizing magnetic, electrical resistivity tomography (ERT), and transient electromagnetic (TEM) surveys—was employed to characterize the Weidenpesch landfill in Cologne, Germany and assess potential groundwater contamination. The results from these methods were consistent, effectively delineating the landfill boundaries and identifying possible contamination. The waste body was distinguished by its relatively low resistivity values with an average value of 1–10 Ω·m in the western and central parts of the landfill and 20–50 Ω·m at its eastern part in contrast with the surrounding high-resistivity gravelly sand layer (several hundreds of Ω·m), and a depth of up to 15 m. The variability in conductivity and magnetic susceptibility across different landfill sections indicated the heterogeneity of buried materials. Additionally, the ERT and TEM data indicate low resistivity values (below 5 Ω·m) at depths of 20–25 m. A correlation with the borehole data suggests that this may represent a contaminated coal/clay layer. Furthermore, repeated TEM measurements revealed significant variation in subsurface conductivity over time, highlighting the need for continuous monitoring. This study demonstrates the effectiveness of an integrated geophysical approach for providing a comprehensive understanding of subsurface landfill conditions, which is essential for informed environmental management and remediation.

## Introduction

Landfills, which serve as repositories for municipal, industrial, and hazardous waste materials, are critical components of modern waste management. However, owing to their complex structure and associated environmental risks, they require sophisticated characterization strategies^1,2^. These sites present a significant global challenge, particularly in Europe, where a large number of old waste sites exist—approximately 90,000 in Germany alone^3^. Contamination of soil and groundwater by landfills poses a significant risk to human health, making it a global environmental issue. Consequently, managing and mitigating these risks is a key focus of sustainable environmental strategies. Most of these old landfill sites in Germany remain unexplored. The Weidenpesch landfill, one such site near Cologne, Germany, is situated just 2.25 km from the Rhine River and raises concerns about potential groundwater contamination. Pollutants could migrate towards the river, threatening both water quality and the surrounding ecosystem. Furthermore, groundwater from this aquifer is used for drinking purposes, emphasizing the need to address these risks. Mitigating these concerns requires detailed subsurface characterization to understand the landfill’s structure and assess its environmental impact.

Traditional landfill characterization methods, such as drilling, sampling, and laboratory analysis, are invasive, costly, and provide limited spatial coverage. Therefore, interest in geophysical techniques has grown due to their non-invasive nature, cost-effectiveness, and minimal environmental disturbance, making them safer alternatives for assessing landfill conditions^4–6^ while enabling a comprehensive understanding of subsurface properties and spatiotemporal dynamics^7,8^. These non-invasive methods are particularly valuable for evaluating legacy landfills, many of which were constructed under standards that did not meet today’s regulations. This has led to serious environmental issues, such as leachate generation and subsequent groundwater contamination^5,9,10^.

Geophysics represents an efficient tool for landfill characterization, providing essential insights into the subsurface composition and material distribution. By employing techniques such as electromagnetics, electrical resistivity, and magnetics, researchers and environmental experts can indirectly detect and interpret physical property variations beneath the landfill surface. These methods facilitate the identification of buried waste, delineation of landfill boundaries, and evaluation of subsurface conditions^10–14^. ERT is extensively utilized in landfill site investigations because of its high sensitivity to variations in soil moisture, clay content, and contaminants^6,15–19^. It is particularly effective in detecting leachate plumes, assessing the integrity of landfill covers, identifying other subsurface irregularities, and evaluating remediation processes^8,20–23^. TEM methods complement ERT by evaluating subsurface conductivity of deeper levels and can provide detailed information on groundwater aquifers, leachate plumes, and potential contamination zones^11,24–27^. These methods are especially valuable because of their ability to define the spatial and vertical extent of subsurface features and monitor temporal changes^11,14^. Magnetics, on the other hand, plays a significant role in landfill characterization due to its ability to detect variations in the Earth’s magnetic field caused by buried metallic objects. These objects, if corroded, can present serious environmental hazards. By conducting magnetic surveys, these metallic objects can be accurately located, and areas with varying magnetic properties can be delineated^5,8,20^. This enables targeted remediation efforts and enhances overall landfill safety. However, detecting shallow anomalies using a single geophysical method is problematic because of the complex and heterogeneous nature of the subsurface. Moreover, individual geophysical methods are inherently susceptible to issues of non-uniqueness.

Our project comprises the first geophysical investigation conducted at the Weidenpesch landfill. Within this project classical radiomagnetotelluric (RMT) and controlled-source radiomagnetotelluric (CSRMT) measurements have been carried out at the Weidenpesch site. Smirnova et al.^28^ found, using CSRMT data, that the waste body exhibits high conductivity (1–10 Ω·m) with a thickness of approximately 7 m. Beneath this, they identified a more resistive layer of gravel and sand (around 100 Ω·m), which is overlaid by a 1 m-thick resistive layer of fine silty sand (up to 1000 Ω·m). Additionally, Fadavi et al.^29^ reported through RMT and CSRMT data that the conductive waste body extends to a maximum depth of 10 m, underlain by a gravelly sand layer extending to about 18 m, which is followed by a conductive brown coal layer.

Moreover, two nearby landfills were also investigated earlier using geophysical surveys. A joint RMT and TEM survey was conducted by Tezkan et al.^30^, along a profile over an old waste deposit in Ossendorf, Cologne, situated 3.5 km southwest of the Weidenpesch landfill. The survey revealed the lateral and vertical extent of the Ossendorf waste site, showing that the waste body has a resistivity range of 1 to 20 Ω·m and extends to a depth of 20 m. In a related study, Ibraheem et al.^5^ employed magnetic and ERT methods to characterize a landfill located 3.5 km west of the Weidenpesch dumpsite. Their study successfully mapped the soil cover as well as the spatial boundaries of the landfill. The waste body has resistivity values below 10 Ω·m and extends down to 13.5 m. They also identified potential leachate plume migration pathways. It is worth noting that the three landfills share similar structures and are hosted in comparable geological formations.

In this research, multiple geophysical methods - magnetics (for detecting metallic objects), ERT (for shallow to intermediate exploration depths), and TEM (for intermediate to deep exploration depths) - were combined with site-specific data, including hydrogeological information, historical records, and borehole data, to enhance the characterization of the Weidenpesch landfill. This novel integrated approach can improve accuracy, reliability, and efficiency by increasing spatial coverage, reducing uncertainties, and offering a cost-effective solution for optimizing waste management strategies, improving remediation efforts, and protecting both the environment and public health.

## History, geology, and hydrogeology of the Weidenpesch landfill

**Fig. 1:**
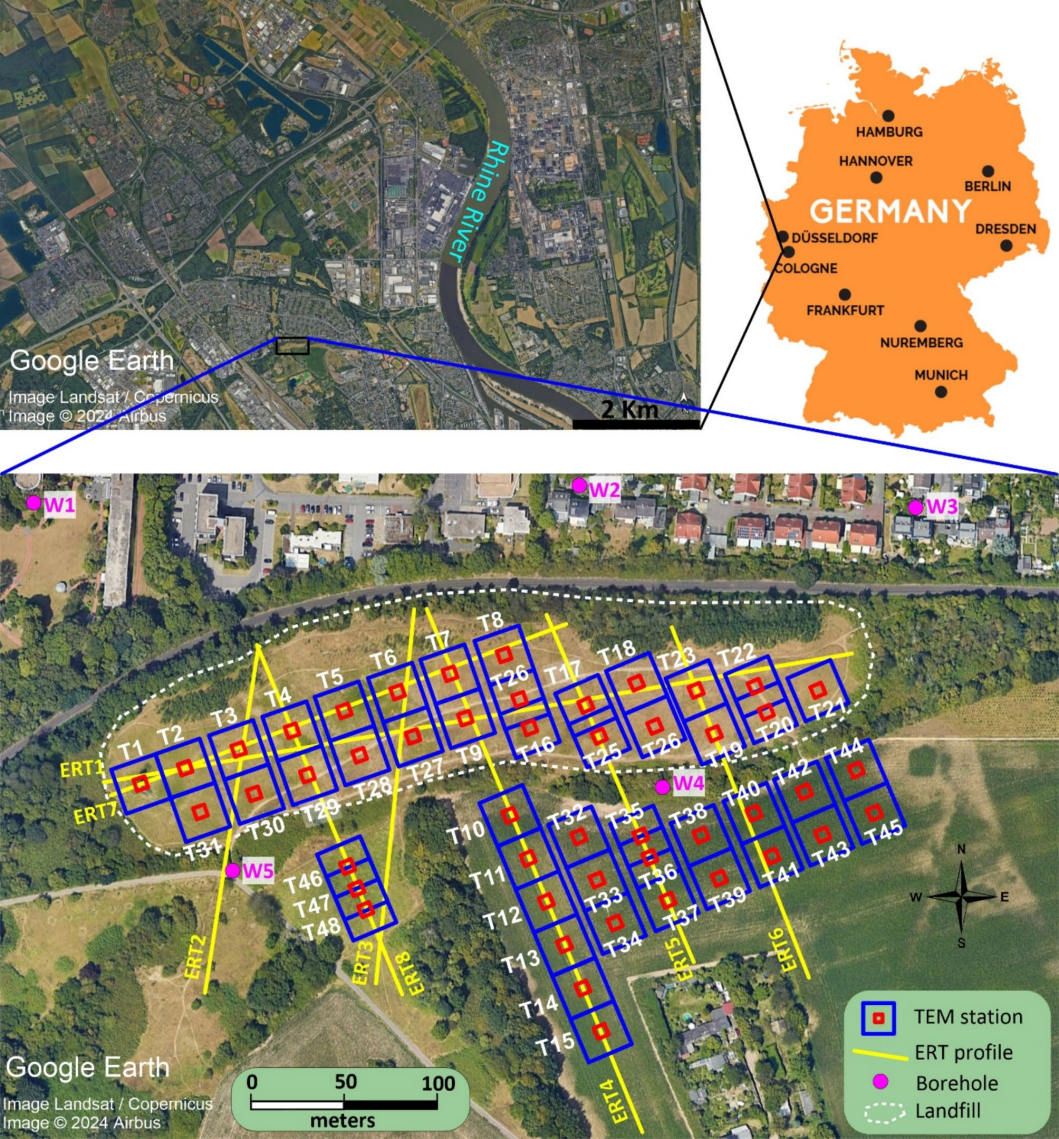
A satellite image depicting the location of the Weidenpesch landfill in Cologne, Germany, illustrating the spatial distribution of the ERT profiles (yellow), TEM stations (blue for transmitter and red for receiver), and boreholes (magenta). The northern boundary of the site is densely occupied by trees, buildings and infrastructure, making geophysical measurements in this part impossible. Maps were created using Google Earth Pro, version 7.3.6.9796 (https://www.google.com/earth/about/versions/). Map data: Google, ©2024 Airbus. Used in accordance with the Google Maps/Earth terms of service for research purposes.

The Weidenpesch waste site located near Cologne, Germany (Fig. 1), originally designated as a sand and gravel pit in the late 1950s, experienced a significant transformation starting in 1966 when it began to serve as a disposal site for various domestic and industrial wastes, including soil material, construction debris, household waste, and dust of grinding stone materials. By its closure in 1976, the site was covered with an irregular thin layer of silty fine sand, averaging 1.5 m in thickness^31^.

**Fig. 2:**
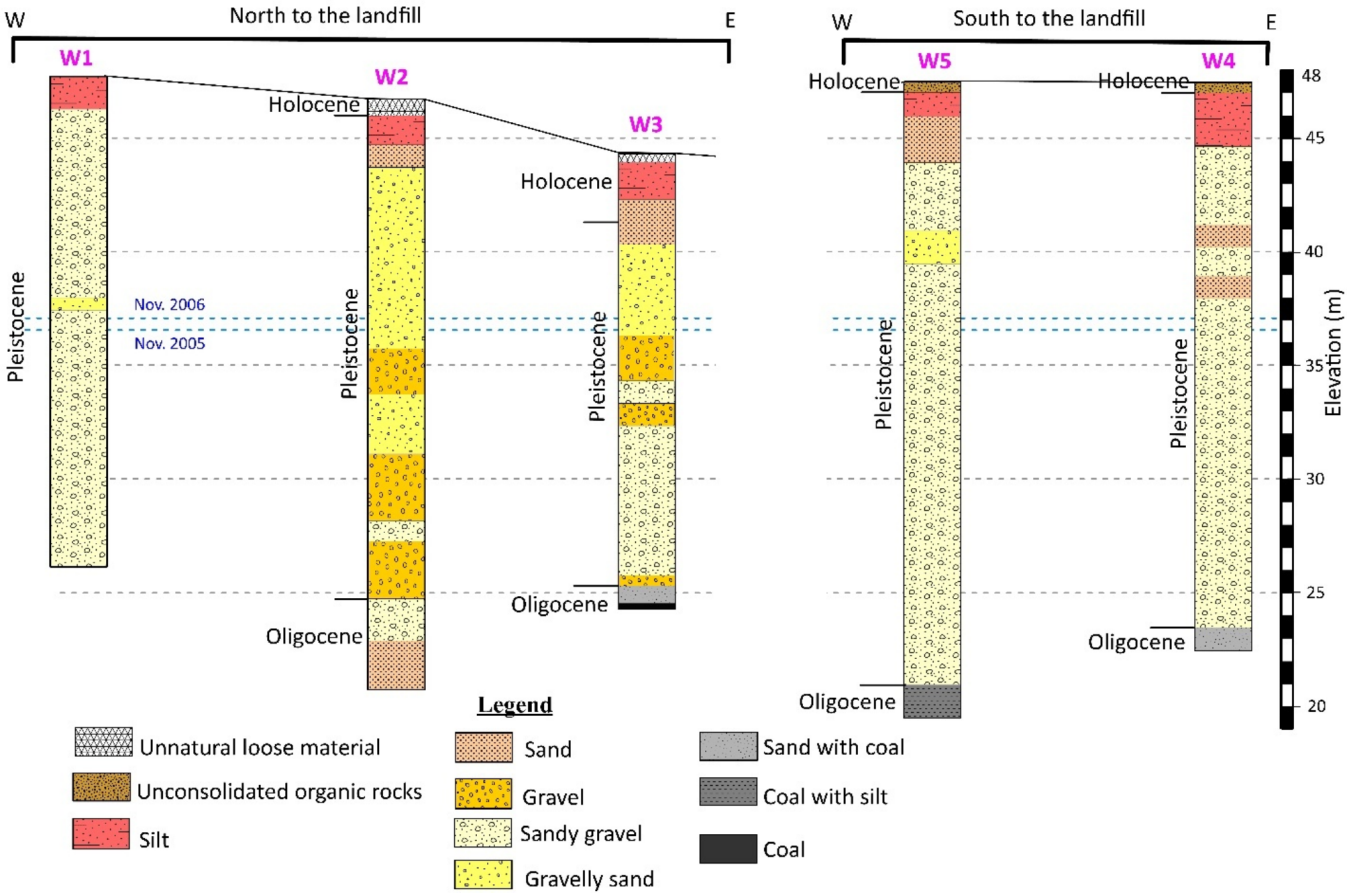
Subsurface lithology logs obtained from boreholes in the area surrounding the Weidenpesch waste site (reproduced after^33,35^). For the locations of these boreholes, refer to Fig. 1. Dashed blue lines represent the groundwater tables in 2005 and 2006.

A geological cross section approximately 1.5 km east of the site revealed that the subsurface consisting of Pleistocene/Holocene floodplain fines overlies a Pleistocene gravelly sand layer, with Tertiary deposits at the base comprising clay, brown coal, and sand^32^. Moreover, subsurface lithology logs (Fig. 2) from boreholes (see Fig. 1) in the undisturbed areas surrounding the landfill indicate that the Holocene layer, consisting of silt, clayey silt, or sandy silt, reaches a thickness of up to 2.8 m. Meanwhile, the Pleistocene gravel and sand layers vary in thickness from 17 m to 25 m. The Oligocene base layer consists of fine sand, sandy coal, silty coal, or brown coal^31–33^.

The geological framework of the area is characterized by a regional depression, infilled predominantly with unconsolidated sediments derived from the River Rhine since the onset of the Tertiary period. These sediments are deposited in alternating sequences of coarse and fine-grained materials, resulting in a multi-aquifer system^34^. For the present study, focus is restricted to the upper aquifer and the underlying clay and coal layers, which exhibit low hydraulic conductivity^32^.

Groundwater level fluctuations in the region are influenced by both seasonal precipitation and the proximity to the River Rhine, located 2.25 km to the east of the study site. Consequently, the river plays a significant control on groundwater dynamics. Groundwater levels at the site exhibited a variation of 3.8 m over a seven-year observation period (1976–1983).At a location 6 km further from the Rhine, the seasonal variation was reduced by approximately one-third, measuring 2.5 m. During the same period, the River Rhine exhibited fluctuations in water level ranging from 36 m to 44 m above mean sea level^32^. Measurements of groundwater levels at boreholes W1–W5 over the course of one year (Table 1) indicate that fluctuations averaged 0.38 m^35^. The groundwater flow measurements highlighted fluctuations in direction over time between northwest and northeastern directions depending on the season^31,35^. Two groundwater flow directions were reported: north-northwest in the western part of the study area and northward in the eastern parts during higher groundwater levels from 1987 (elevation of 38.40 m). For lower groundwater levels (37.1 m), a northwest flow direction was observed in 1983. The hydraulic conductivity for the gravel layer was estimated to be around 5 × 10⁻³ m/s, and the groundwater flow velocity was relatively slow, at 1.12 m/day due to the small difference in the observed groundwater levels^31^.

**Table 1:** Groundwater levels at boreholes W1–W5 during 2005 and 2006^35^.

Borehole	Water level (elevation in m)		Difference (m)
	November 2005	November 2006	
W 1	36.70	37.08	0.38
W 2	36.65	37.07	0.42
W 3	36.60	37.03	0.43
W 4	36.71	37.07	0.36
W 5	36.77	37.10	0.33
Average	36.69	37.07	0.38

### Geophysical field surveys and data acquisition

To better characterize the Weidenpesch dumpsite, we employed an integrated approach that combined three distinct geophysical techniques along with available historical, borehole and subsurface geological information. The geophysical data acquisition methods used at the site are described below.

### Magnetic survey

**Fig. 3:**
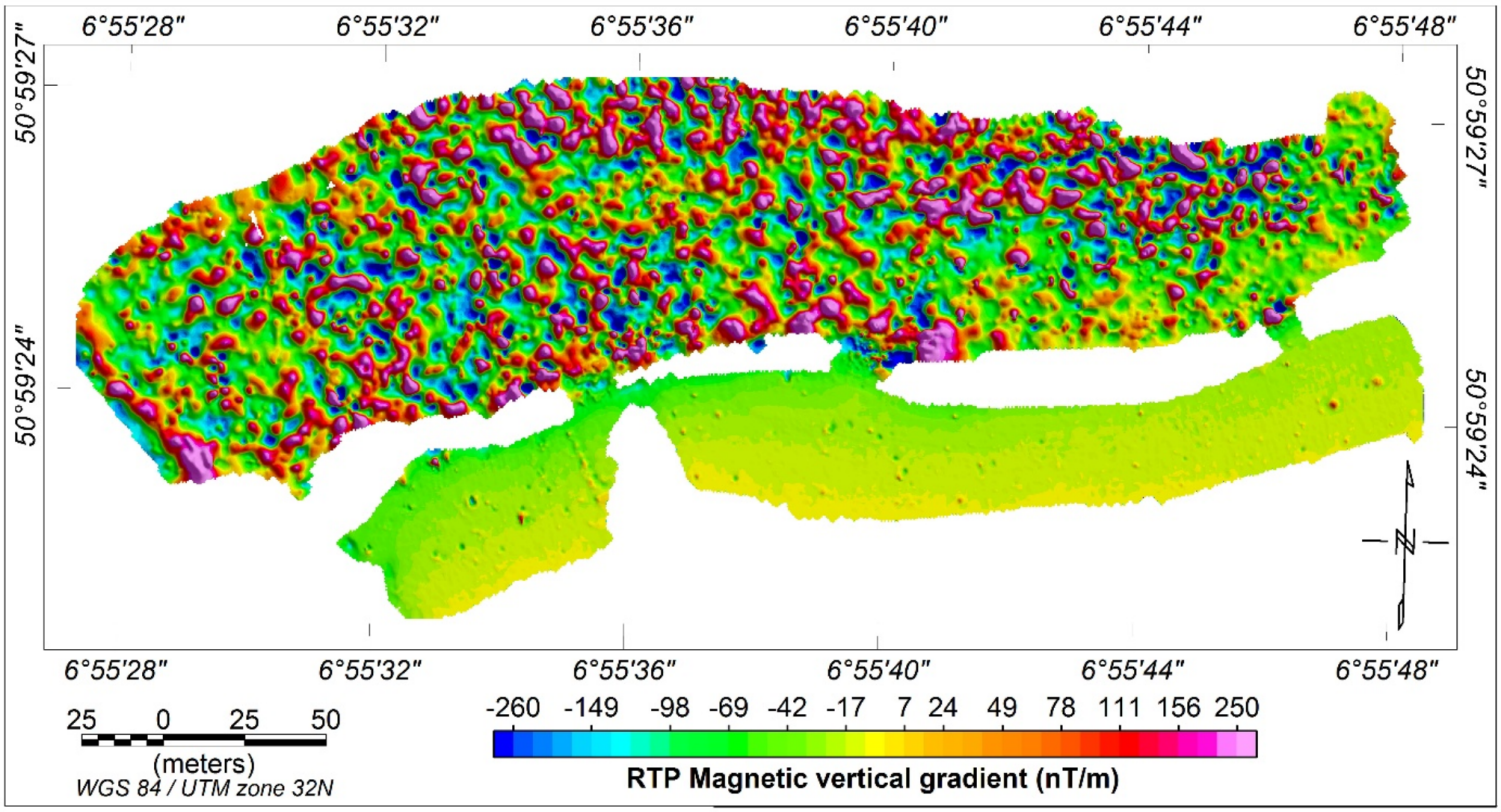
The RTP magnetic vertical gradient map of the Weidenpesch landfill. Red and red/blue colours refer to highly magnetic source materials dumped in the landfill.

A ground magnetic survey was carried out using a GEM Systems GSM-19T proton magnetometer in gradient mode, which employs two vertically aligned magnetic sensors to automatically correct for diurnal variations in the Earth’s magnetic field. The coordinates, time, and elevation of each magnetic reading were simultaneously recorded with the magnetometer’s high-quality internal GPS system. Magnetic measurements were continuously collected in gradient walking mode at a 1-second sampling rate, forming a grid with approximately 1 m ×1 m spacing, to detect metallic materials both within and outside the landfill. The survey was completed over a period of five days. The acquired magnetic data were gridded using the minimum curvature method and then transformed to the wavenumber domain using Fast Fourier Transform (FFT). To correct for the inclination of the Earth’s magnetic field and reposition magnetic anomalies over their sources, the magnetic data were reduced to the north magnetic pole (RTP)^36^ using inclination and declination values of 66.35° and 2.58°, respectively. This process yielded a RTP magnetic vertical gradient map (Fig. 3), which clearly delineates the horizontal boundaries of the dumpsite. Additionally, the RTP magnetic map reveals various distinct magnetic zones within the area, indicating different types of waste. Some local magnetic anomalies detected outside the waste site suggest the presence of metallic bodies in the shallow subsurface.

### ERT survey

This technique involves placing a series of electrodes on the ground to inject electrical current and measure the resulting voltages, enabling the mapping of subsurface properties along 2D profiles of apparent electrical resistivity. The data are then typically processed via an inversion routine to obtain true resistivity values^6,37,38^. Eight ERT profiles (Fig. 1) were designed and measured to cover most of the landfill area. The ABEM Terrameter LS resistivity meter with 64 electrodes and 4 cables was utilized. To extend some of the ERT profiles, a roll-along technique was employed, which involved shifting a portion of the electrode layout in the desired direction. Both the Wenner and dipole-dipole configurations were used for each ERT profile, with electrode spacings of 2.5 m in the second and third cables and 5 m in the first and fourth cables. The reason for choosing these arrays is that the Wenner array is particularly effective for detecting horizontal structures and provides good vertical resolution, whereas the dipole-dipole array excels at resolving lateral variations in resistivity and is sensitive to vertical structures^21,39^. The resistivity data obtained from these different arrays were combined into a single dataset to form a mixed array. This approach enhances subsurface data coverage, increases sensitivity, and provides good lateral and vertical resolution^5,40^.

The L1-norm regularization technique was used to invert the resistivity data, minimizing the absolute differences between the measured and calculated apparent resistivity values through an iterative process, where the accuracy of the data fit is expressed in terms of the absolute error^41^. The L1-norm inversion technique was chosen for its ability to produce a sharper contrast between the resistivity values of the waste body and the surrounding medium, outperforming the L2-norm method in this regard. Additionally, the L1-norm technique is less sensitive to outliers or bad data points, making it a more robust option compared to the L2-norm method. As a result, the L1-norm inversion yields models with sharper edges and generally provides better imaging results than the L2-norm^5,42^. The optimization is given by Eq. (1)^38^:

1 $$\begin{aligned} & (J^{T} R_{d} J + \lambda (\alpha _{x} C_{x}^{T} R_{m} C_{x} + \alpha _{y}C_{y}^{T} R_{m} C_{y} + \alpha _{z} C_{z}^{T} R_{m} C_{z} ))\Delta q_{k} = J^{T} R_{d} g -\lambda( {\alpha _{x} C_{x}^{T} R_{m} C_{x} } \\ & \quad  { + \alpha _{y}C_{y}^{T} R_{m} C_{y} + \alpha _{z} C_{z}^{T} R_{m} C_{z} )q_{k} }  \end{aligned}$$

where $J$ is the Jacobian matrix of partial derivatives; $\lambda$ is the damping factor; ${R}_{d}$ and ${R}_{m}$ are the weighting matrices; ${C}_{x}$, ${C}_{y}$ and ${C}_{z}$ are the roughness filter matrices in the x-, y- and z-directions; ${\alpha}_{x}$, ${\alpha}_{y}$, and ${\alpha}_{z}$ are the relative weights given to the roughness filters; $g$ is the discrepancy vector; and ${\varDelta\;q}_{k}$ is the model update vector in the K-th iteration of model parameter $q.$

Information about the ERT profiles, including profile lengths, electrode spacings, number of iterations, and absolute errors, can be found in Table 2.

**Table 2:** Characteristics of ERT profiles.

Profile	Length (m)	Electrode interval (m)	Iteration number	Abs. error %
ERT 1	400	2.5	6	3.13
ERT 2	185	2.5	8	2.37
ERT 3	200	2.5	7	3.82
ERT 4	300	3.0	10	1.40
ERT 5	200	2.5	11	1.24
ERT 6	200	2.5	10	1.54
ERT 7	260	2.5	6	2.18
ERT 8	200	2.5	9	1.4

### TEM survey

TEM uses a transmitter loop on the surface to generate a controlled primary electromagnetic field. When the current in the transmitter loop is abruptly turned off, eddy currents are induced in the subsurface, producing a secondary magnetic field. This field is measured at the surface by a receiver loop, and the decay of the induced voltage over time is analyzed to infer subsurface electrical conductivity^3,43^. A total of 48 TEM stations were measured inside and outside the landfill using a central loop configuration (Fig. 1). Additionally, noise measurements were carried out, which revealed that the measured transients were above the noise level, especially in the time range of 1–10 ms. Inside the landfill, the transmitter loop size was 25 × 25 m^2^, and the receiver loop size was 5 × 5 m^2^, whereas outside the landfill, the receiver loop size was 10 × 10 m^2^. The Zonge GDP-3224, an integrated, 24-bit multichannel receiver for acquiring controlled- and natural-source geoelectric and electromagnetic data, was utilized. The system provides two acquisition modes with different exploration depths. The current used was 3 A for the nanoTEM (time range: 1.5 × 10^−3^ − 1 ms) and 10 A for the zeroTEM (time range: 0.1–6 ms) measurements. The transients from NanoTEM and ZeroTEM were merged for each station to form one transient, with the ramp time of the NanoTEM data (2.7 µs) considered for the inversion process. The TEM data were inverted using the Emuplus 1D inversion code developed by the University of Cologne. This code allows for 1D forward and inverse modeling of TEM data. The data fit (χ) is calculated via Eq. (2):

2 $$\chi=\sqrt{\frac{1}{N}\sum_{i=1}^{N}{\left(\frac{{d}_{i}-{{d}_{i}^{{\prime}}}}{\delta\;{d}_{i}}\right)}^{2}}$$

where ${d}_{i}$ and ${d_{i}^{\prime}}$ represent the measured and calculated data, respectively. The term $\delta\;{d}_{i}$ denotes the data error, and N is the total number of data points. An optimal fit is achieved when $\chi=1$ which means that that the difference between the measured and the calculated data equals the data-error. A $\chi$ value less than 1 indicates overfitting, while a value greater than 1 suggests that the data are not sufficiently fitted^44^.

## Results and discussion

The integrated application of magnetic, ERT, and TEM methods at the Weidenpesch landfill provided a comprehensive perspective on subsurface characterization, with each method complementing the others to create a more complete understanding of the site.

### Results of the magnetic survey

The magnetic survey, which utilized high-resolution RTP magnetic vertical gradient mapping, effectively delineated the landfill boundaries and detected distinct zones of varying magnetic properties, indicative of different types of buried waste (Fig. 3). This method was particularly useful in identifying metallic objects within and outside the landfill’s assumed boundaries, which could pose serious environmental risks due to potential corrosion.

**Fig. 4:**
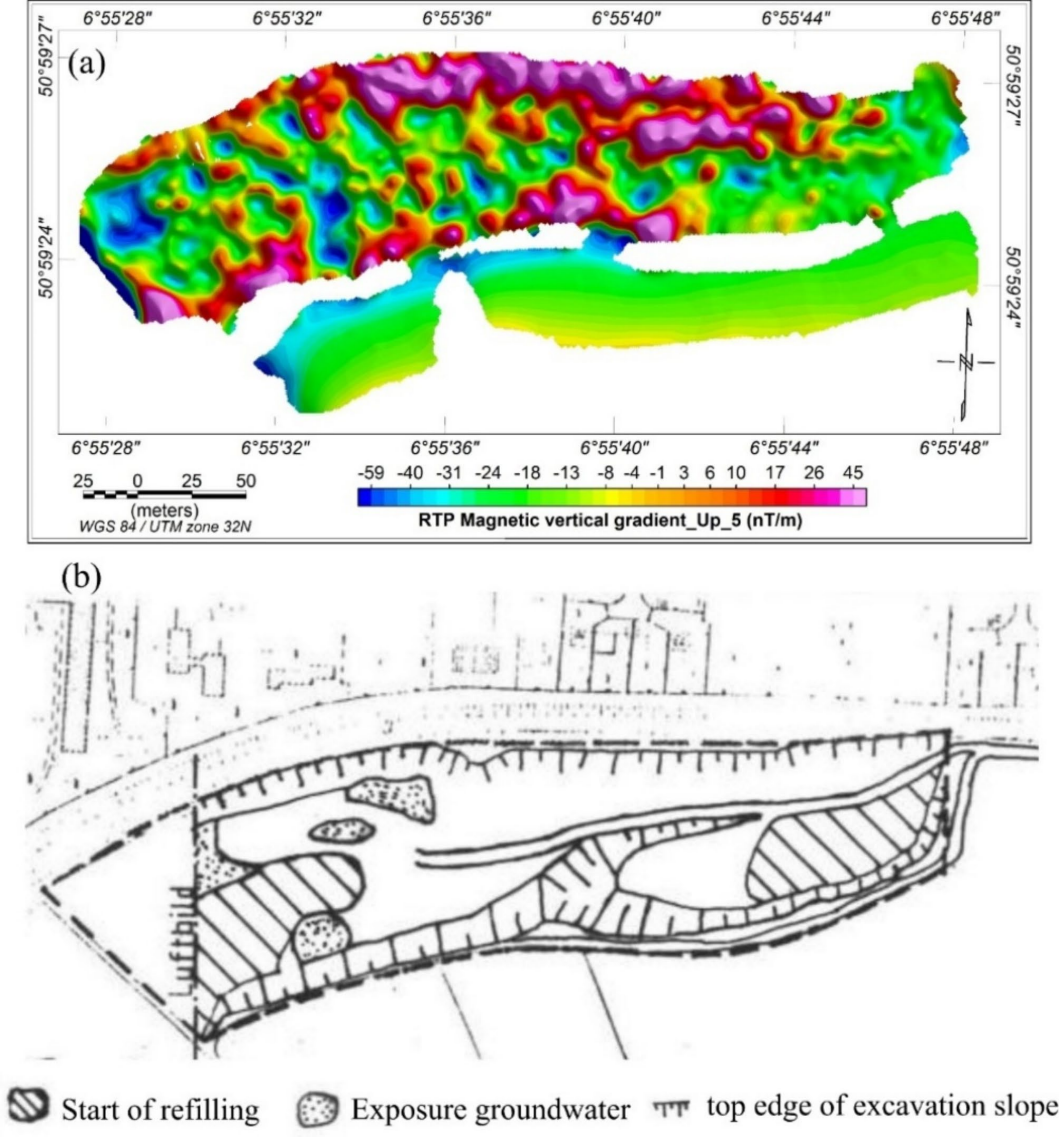
(**a**) The RTP magnetic map after applying an upward continuation filter at an altitude of 5 m, and (**b**) a historical map depicting the status of the Weidenpesch landfill in 1966. The refilling may consist of dust from grinding stone materials^31^.

To reduce the influence of near-surface metallic objects, an upward continuation filter was applied to the RTP magnetic data, extending to a height of 5 m, as depicted in Fig. 4a. A comparison with a historical map from 1966, which details the landfill’s condition at that time, revealed a good correlation. The resulting map allowed for the differentiation of several zones: a central zone predominantly representing areas with relatively high metal contents, and eastern and western zones where nonmagnetic materials are present (refilling areas, Fig. 4b). Historical records suggest that these nonmagnetic materials may include dust of grinding stones, other stone-debris, and household waste. Although the magnetic survey effectively identified and mapped the distribution of metallic materials, it provided limited insight into the non-metallic components and the overall geometry of the waste body.

### Results of the ERT survey

**Fig. 5:**
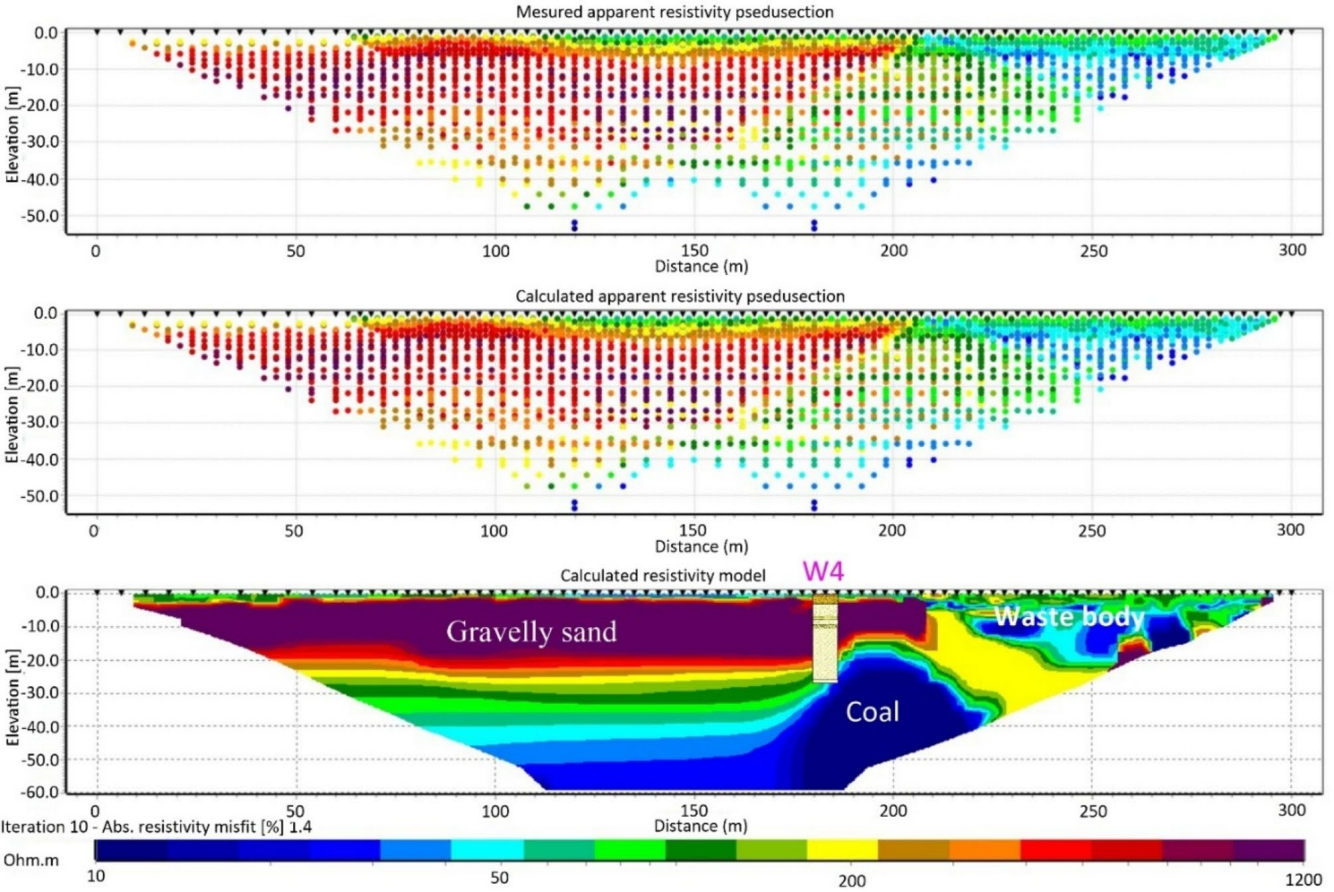
Measured and calculated apparent resistivity pseudosections, along with the inverted resistivity 2D model obtained using robust inversion (L1-norm) of the ERT data of profile ERT4 after 10 iterations (absolute error = 1.4%). The borehole log from W4 is superimposed on the 2D cross section.

The ERT survey offered detailed insights into the subsurface resistivity structure, clearly distinguishing between the low-resistive waste body and the surrounding high-resistive gravelly sand layer. Figure 5 illustrates the measured and calculated apparent resistivities and the inverted 2D resistivity model of profile ERT 4. The data fitting for this profile has an absolute error of 1.4%. For the other ERT profiles, the misfit ranges from 1.24% in Profile ERT5 to 3.82% in Profile ERT3, as shown in Table 2. The 2D model correlates well with the W4 borehole log outside the landfill (Fig. 5), successfully identifying the gravelly sand, coal, and waste body. Within the landfill, the coal layer was not detected along this profile.

**Fig. 6:**
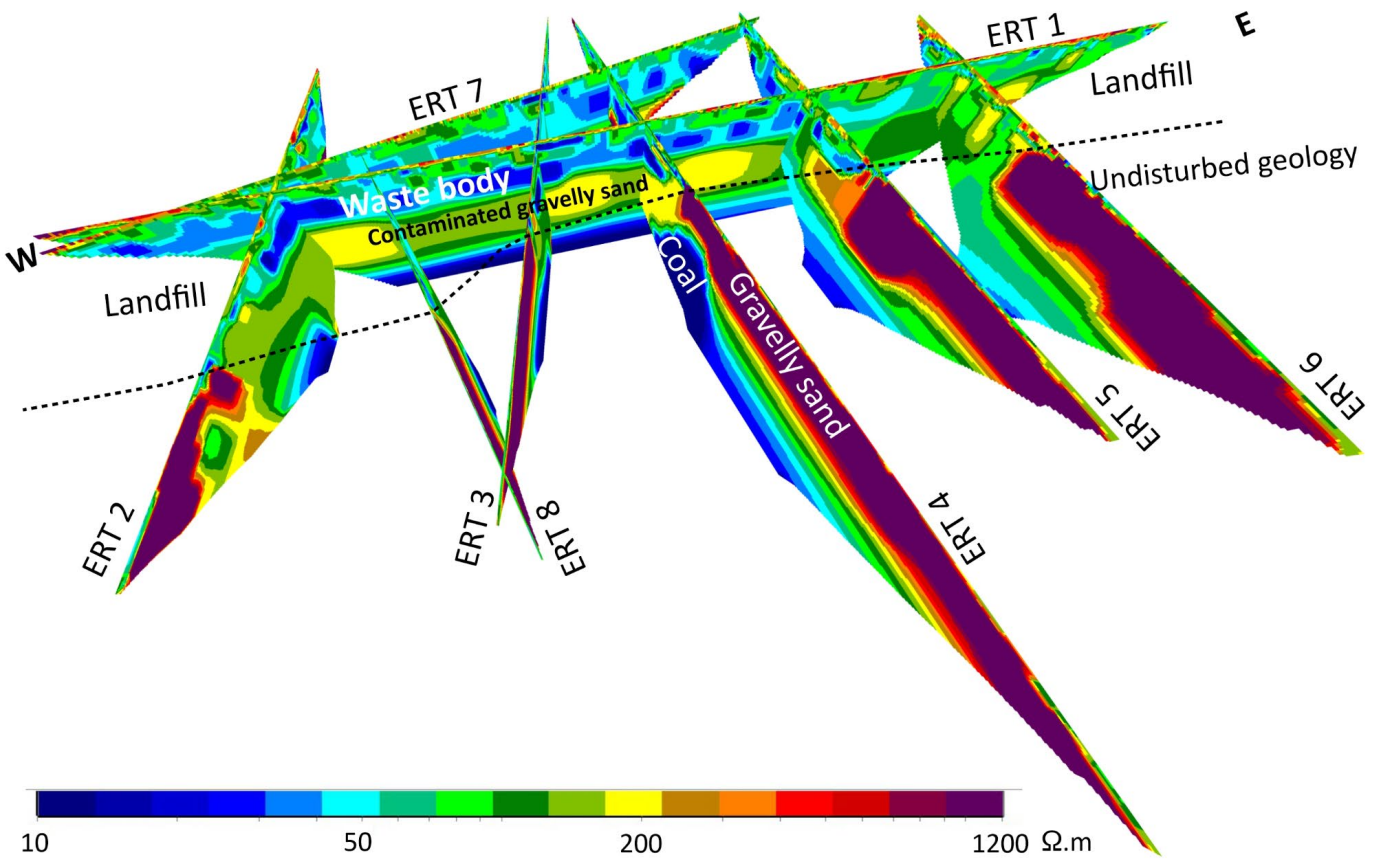
A 3D fence diagram depicting the subsurface resistivity distribution derived from the 2D inversion of ERT data. The blue anomalies at shallow depths represent the waste body. The dashed black line represents the southern boundary of the landfill.

Additionally, the resistivity of the gravelly sand layer beneath the waste body dropped significantly, from over 1200 Ω·m to about 200 Ω·m. The 2D cross sections from all ERT profiles were combined to create a 3D fence diagram (Fig. 6). The 3D model reveals that the waste body extends to varying depths across the landfill, reaching up to 15 m in some locations, which exceeds the depths reported by Smirnova et al.^28^ and Fadavi et al.^29^. In contrast to the eastern section, the resistivity in the western and central areas is lower, likely due to the heterogeneous composition of the buried materials. An average resistivity value of 50 Ω·m was determined for the waste body in the eastern part of the landfill, while the central and western parts showed an average value of 10 Ω·m.

The coal layer is detected at a depth of 20 m along profile ERT 4, between distances of 190 m and 205 m, and then dips toward the north. This approach was particularly effective in identifying potential contamination beneath the landfill. For instance, a layer with lower resistivity values compared to its resistivity values in the undisturbed geology outside the landfill, detected at depths below 10–15 m, correlates with borehole data and suggests the presence of a contaminated gravelly sand layer. The resistivity values of this layer have decreased from over 1200 Ω·m outside the landfill to around 100–200 Ω·m within it, likely due to the increase in ionic concentrations resulting from the downward leaching of contaminants, especially as the dumped materials are in direct contact with the groundwater of the shallower aquifer. Although the ERT method provided superior vertical resolution and precision in detecting subsurface anomalies, it was less effective in detecting highly localized metallic objects compared to the magnetic survey. Additionally, the resolution of the ERT method decreases at greater depths.

### Results of the TEM survey

**Fig. 7:**
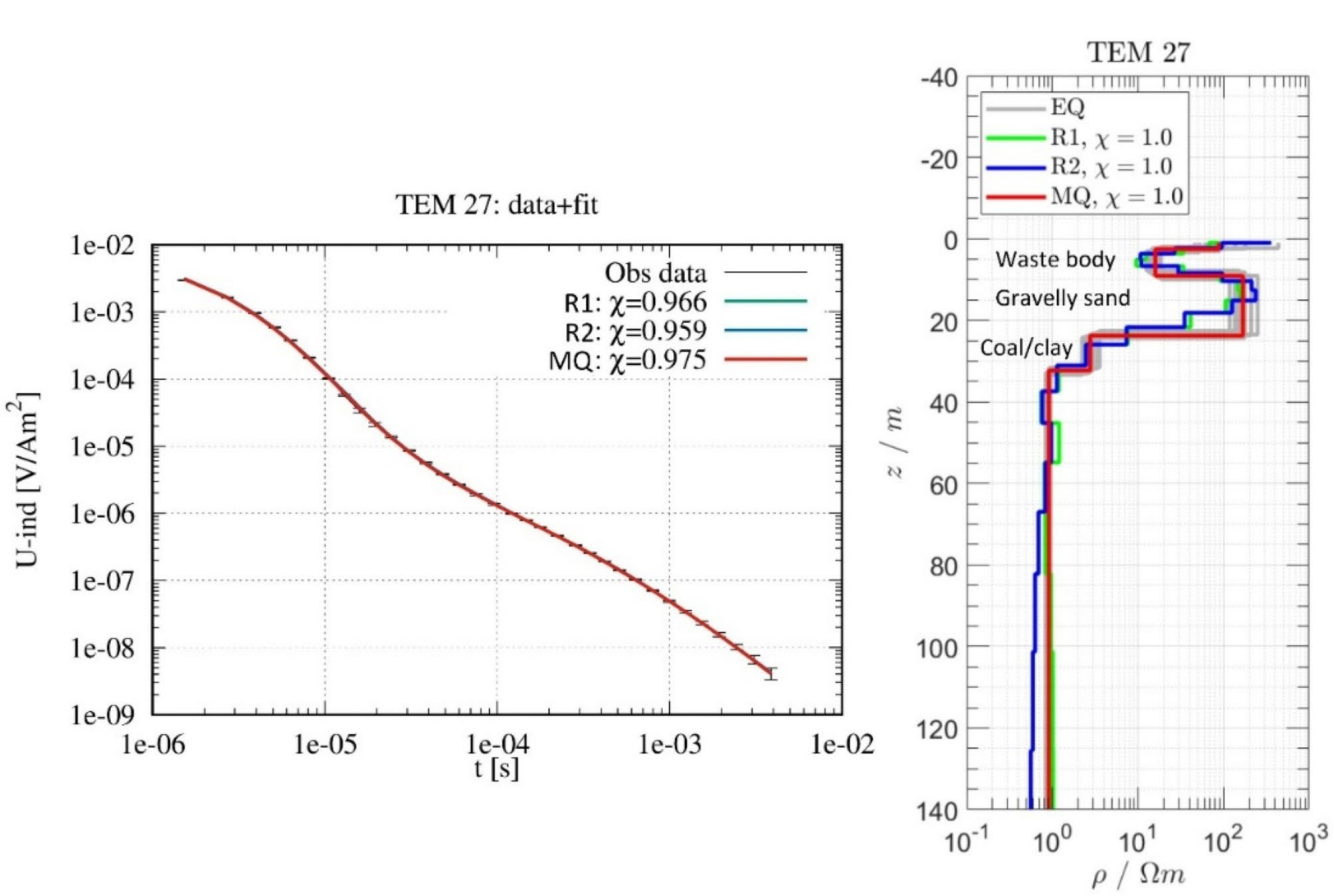
1D inversion results for station TEM27. The MQ model is denoted in red, while the Occam models with different roughness values R1 and R2 are shown in green and blue, respectively. The EQ models are displayed in light grey. Data fit (χ) is displayed in the legend of the model.

The TEM survey further enhanced the characterization of the landfill by offering a vertical profile of subsurface conductivity, complementing the horizontal imaging provided by ERT to greater depths. Figure 7 presents an example of the 1D inversion and interpretation for station TEM27 (Fig. 1), displaying the results from both Occam with different roughness constraints (R1 and R2)^45,46^ and Marquardt-Levenberg (MQ)^47,48^ inversions, alongside equivalent (EQ) models^26,44^. R1​ denotes first-order differences to prevent abrupt resistivity changes between layers, whereas R2​ uses second-order differences, ensuring a gradually varying model with minimal curvature. EQ models are different resistivity-depth models that fit the observed data equally well. This non-uniqueness in geophysical inversion leads to multiple models fitting the data equally, causing interpretation ambiguity.

At station TEM27, the waste body is identified at a depth of 2.6 m with a thickness of 6.5 m overlaid by a soil cover. This thickness value aligns with the results reported by Smirnova et al.^28^. Below this waste body, a resistive layer, indicative of gravelly sand, extends down to a depth of approximately 23.5 m. This is followed by a highly conductive layer, interpreted as wet lignite (brown coal) or clay, on the basis of a comparison with geological data and ERT results (Figs. 2, 5 and 6).

**Fig. 8:**
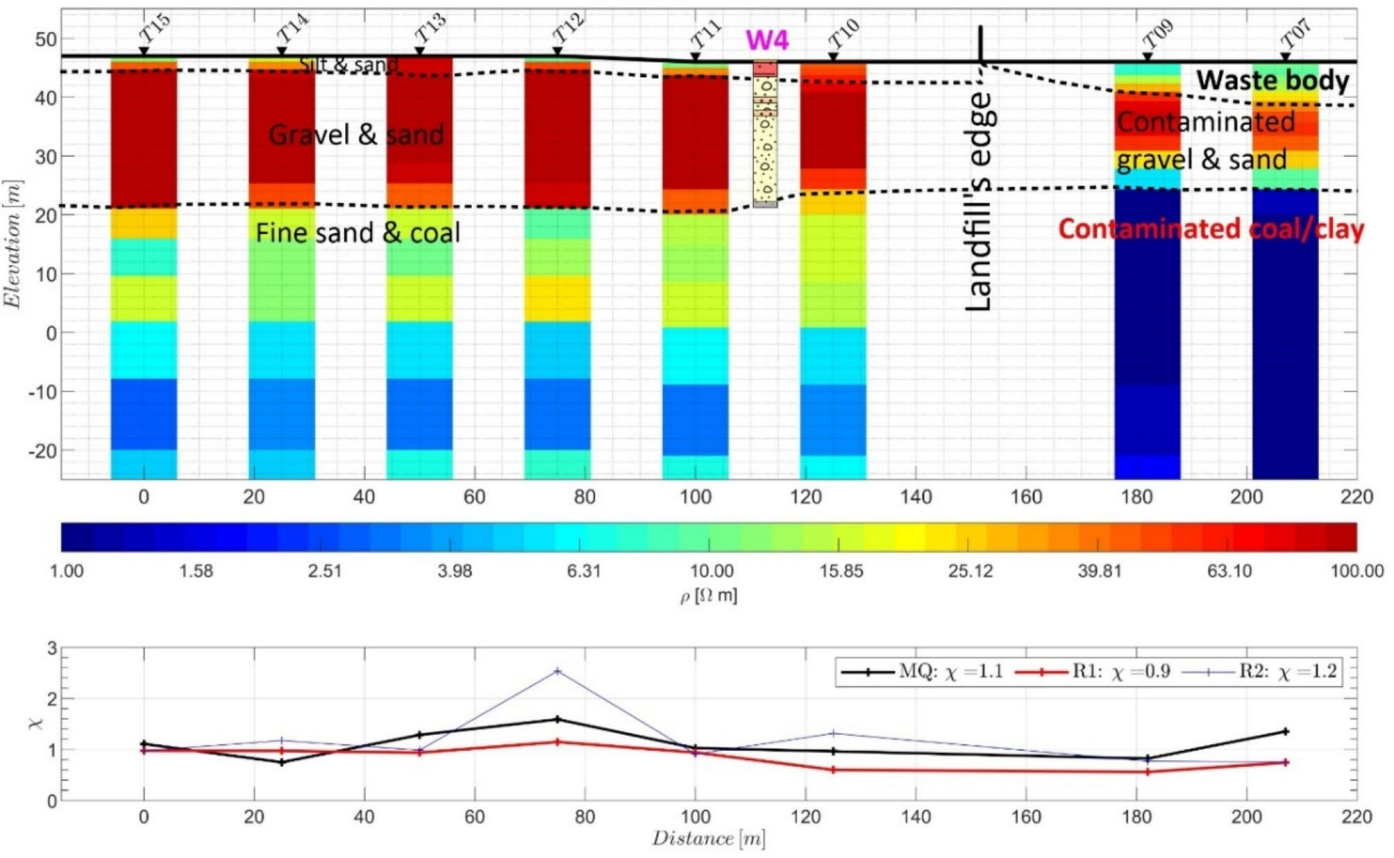
2D resistivity cross section obtained from the 1D Occam inversion results with roughness parameter R1 for the TEM data along profile ERT4 (see Fig. 1). The lower panel displays the χ values for the Occam (R1 and R2) and MQ inversions. The log of borehole W4 is presented between TEM stations T10 and T11.

Figure 8 presents a 2D cross section interpretation of eight TEM soundings (TEM07, TEM09, TEM10, TEM11, TEM12, TEM13, TEM14, and TEM15) along profile ERT4 (refer to Fig. 1 for the locations of the stations) obtained from the Occam R1 inversion results. As expected, the resistive soil cover is not detected within the landfill by the TEM method. However, the TEM data successfully identified three layers within the landfill. The first layer, with resistivity values ranging from 5 to 20 Ω·m, corresponds to the waste body with a thickness of approximately 7 m below station TEM7, consistent with the value observed by Smirnova et al.^28^. The second layer, with a resistivity of approximately 80 Ω·m, represents the gravelly sand layer. The third and most conductive layer, with a resistivity of approximately 1 Ω·m, is identified as the wet coal/clay layer and is well correlated with borehole W4. A comparison of the resistivity values inside and outside the landfill, particularly for the gravelly sand and coal/clay layers, reveals a significant reduction, which may indicate contamination leakage from the waste body downward. Three distinct layers were also detected outside the landfill. The first layer, with a thickness of 2–3 m, represents the Pleistocene/Holocene floodplain fines; the second layer, consisting of Pleistocene gravelly sand, has a thickness of 20–24 m; and the base of the section is composed of Tertiary deposits, including clay/coal. These findings are consistent with ERT results and the available geological information and borehole data (refer to Fig. 2). It is worth mentioning that the TEM technique provided better results than the ERT technique for detecting the coal layer beneath the landfill.

**Fig. 9:**
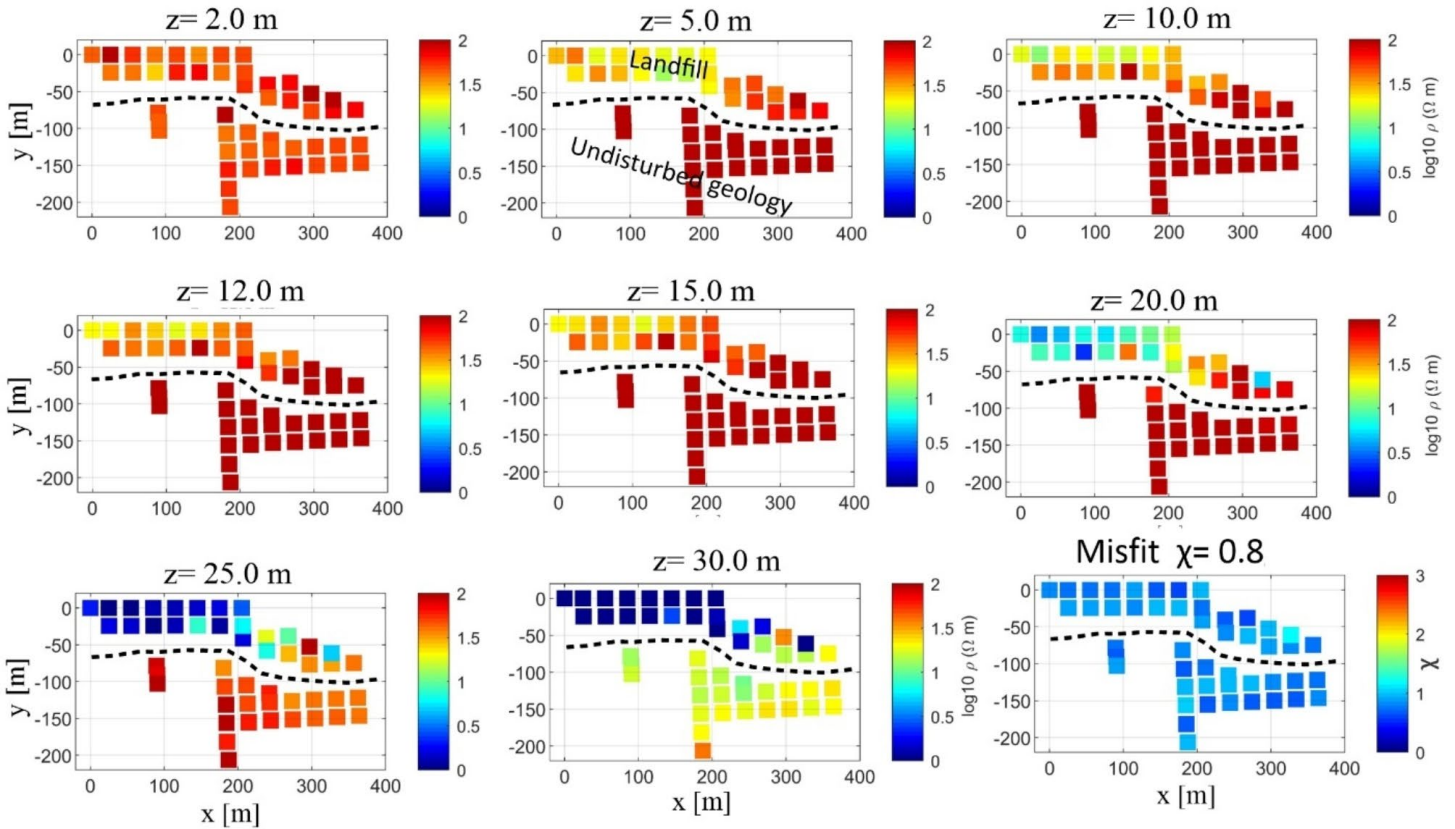
Depth slices at 2, 5, 10, 12, 15, 20, 25 and 30 m obtained from the results of the 1D Occam inversion of the TEM data (Fig. 1). The waste body is observed at depths down to 10 m. Below this depth, a contaminated gravelly sand layer is detected extends to a depth of 20–23 m. The base layer, consisting of saturated coal and clay, is likely contaminated as well, owing to the expected high porosity of the overlying gravelly sand layer. There is a significant contrast in resistivity values at equivalent depths, both inside and outside the landfill, particularly concerning the gravelly sand and coal/clay layers. This discrepancy could indicate potential contamination leachate. The average χ value indicates a good fit.

The 1D Occam inversion results at various depth slices (Fig. 9) from all the TEM stations (Fig. 1) reveal that the waste body extends to depths of up to 10 m. Beneath the landfill, the gravelly sand layer, reaching depths of 20–23 m, likely facilitates the passage of contaminants due to its potential high porosity. This is evidenced by the significantly lower resistivity values of the gravelly sand layer below the landfill compared with its resistivity in the undisturbed geology outside the landfill. The results also suggest possible contamination accumulation, particularly within the coal/clay layer with a resistivity of less than 1 Ω·m especially in the western and central sectors of the landfill, which is expected to have low porosity. Unfortunately, the base of this highly conductive layer was not identified. Additionally, the eastern area of the landfill exhibits higher resistivity values than the central and western areas. The average χ value indicates a good fit, as shown in Fig. 9. These findings align well with the results of the ERT survey. While the ERT survey provided clear imaging of shallow depths, the TEM survey produced more accurate results at greater depths.

The results of the 1D inversion of the TEM data are consistent with the borehole data and ERT results, which helps to validate the overall findings despite the 1D model’s simplicity. While the central loop configuration has a limited footprint around the transmitter, the induced currents are focused beneath this area, minimizing 2D/3D effects^49^. Thus, 1D inversion is suitable for most TEM data interpretations, except at the borders of lateral inhomogeneities (e.g., waste site boundaries) or areas with strong topography. Overall, these effects are rated as minor due to the self-consistency of the 1D models. Additionally, in the central loop configuration, measuring the vertical component of the magnetic field at the centre of the loop helps to reduce the influence of subsurface lateral inhomogeneities^50^.

**Fig. 10:**
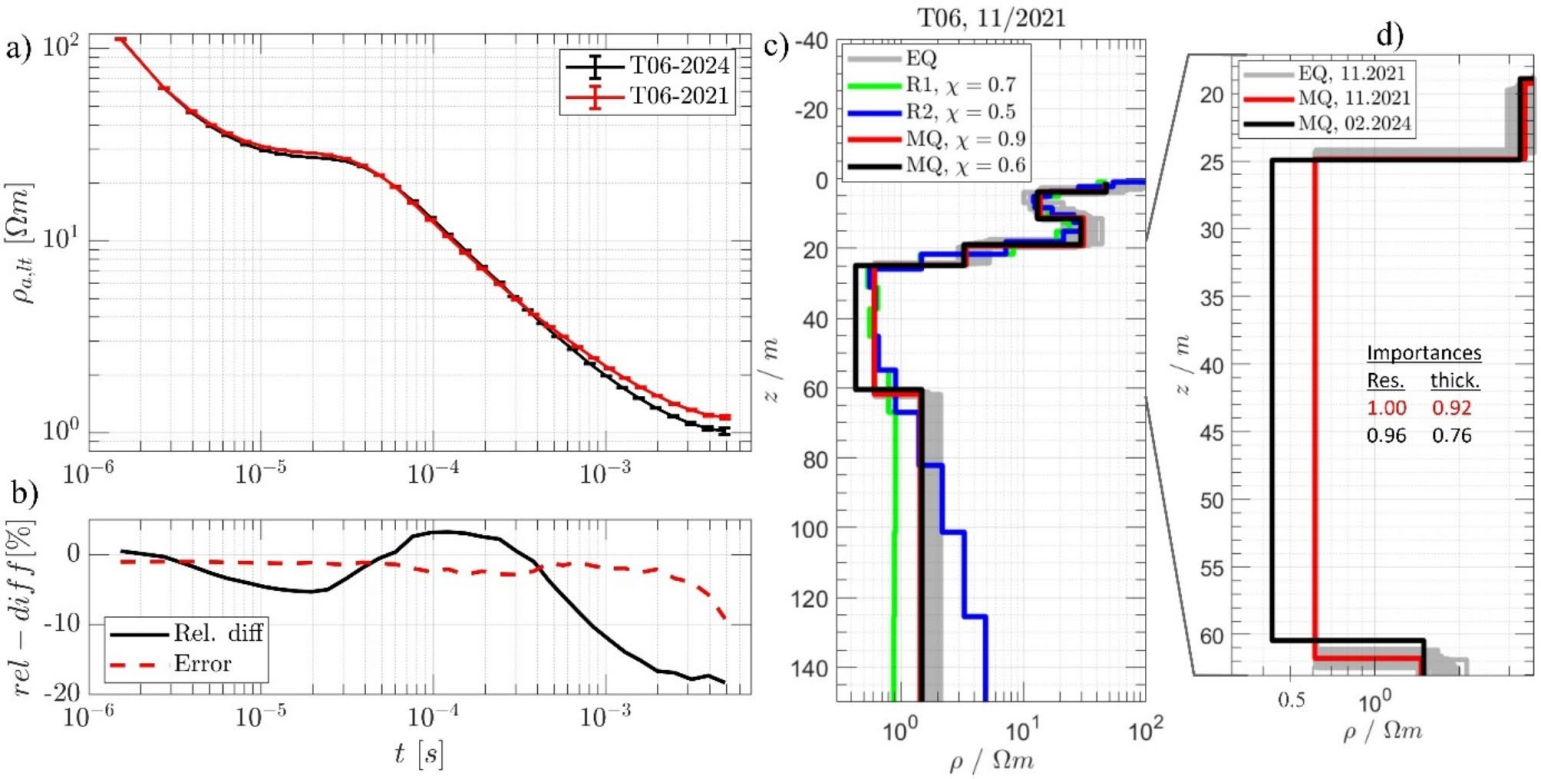
A detailed analysis of TEM station T06, focusing on measurements from November 2021 and February 2024. a) the late-time apparent resistivity approximations where a notable 20% increase in conductivity within the 25–60 m depth range since 2021 is depicted in the relative difference curve (b). c) 1D inversion results. The MQ model from November 2021 is depicted in red, with the Occam models shown in green and blue for different roughness values (R1 and R2), and the EQUI models in light grey. The February 2024 MQ model is shown in black for comparison. The data fit (*χ*) is indicated in the legend. Additionally, the thickness and resistivity importance of the deep conductor, with good importance values determined by the MQ model are highlighted in (d).

To investigate changes in subsurface conductivity beneath the waste body over time, repeated TEM measurements were conducted at station T06. The initial measurement was carried out on November 15, 2021, and a follow-up measurement occurred on February 20, 2024. Figure 10 presents the late-time approximation and 1D inversion results for this station. Focusing on detecting deep conductors at late times, the common transformation was applied to the late-time apparent resistivity (${\varvec{\rho}}_{\varvec{a},\;\varvec{l}\varvec{t}}$). This transformation reduces the dynamic range, providing initial insights into the subsurface structure^51^. As shown in Fig. 10a, significant changes occur at late transient times, with relative differences reaching up to 20% (Fig. 10d), far exceeding the error level. The quality of the MQ inversion results is assessed using EQ models and parameter importances to estimate model uncertainties, with parameters closer to one indicating better resolution^44^. The resistivities and layer thicknesses are generally well-resolved. The discrepancies between the Occam R1 and R2 models suggest an exploration depth of 80 m (Fig. 10b). Overall, the data fitting is optimal, with *χ* values close to one. Particular attention is given to the contaminated coal/clay layer at about 25 m depth, where a clear increase in conductivity over time is observed, as depicted in Fig. 6c. Within the depth range of 25 m to 60 m, the conductivity increases by 34% from 0.41 to 0.62 Ω·m, a significant relative change measurable by the TEM (and also by ERT) method, which is evident in the response change. According to data from the German Weather Service (DWD), the precipitation rate in autumn 2021 was below the long-term average, with September showing only 43% of typical levels at Cologne-Bonn Airport. In contrast, precipitation in winter 2023–2024 was significantly above average, reaching 191% in November^52^. This increase in rainfall may lead to greater washdown of contaminants, which could explain the rise in conductivity. Additionally, the low topography of the landfill, compared to the surrounding area, likely causes rainwater to accumulate, increasing infiltration through waste materials into deeper layers. This process can contribute to leachate production, which may raise the potential environmental impact.

Although the two measurements taken at TEM station T06 in the landfill are insufficient to fully characterize time-dependent changes in subsurface conditions, they do offer valuable insights into the potential sustained migration of pollutants from the waste body over the years. However, to accurately assess temporal variations, such as seasonal fluctuations in the water table, a more comprehensive long-term monitoring program is necessary. This would require expanding the scope of the study by incorporating additional methods and monitoring techniques. Key tasks could include installing a monitoring borehole within the landfill for time-lapse measurements of groundwater levels, hydrochemical properties, temperature, and conductivity. Gathering precipitation data and employing time-lapse ERT and TEM surveys would also be essential for tracking potential contamination over time, especially during periods of increased rainfall. These steps would provide a more precise understanding of how seasonal factors influence the movement of contaminants and help characterize the speed and distribution of leachate in both soil and groundwater.

### Integration of results

The integration of Magnetic, ERT, and TEM results represents a critical aspect of this study, providing a comprehensive understanding of the subsurface characteristics and contamination processes at the landfill. The magnetic survey effectively delineated the landfill boundaries and identified metallic objects that could pose environmental risks, offering precise spatial data for defining areas of interest. However, its inability to provide detailed information about non-metallic components or the overall waste geometry necessitated the inclusion of complementary methods. The ERT survey contributed significantly by offering detailed resistivity profiles that clearly distinguished low-resistive waste materials from high-resistive gravelly sand layers. It also identified zones of contamination, such as ionic leachates, extending below the waste body into the gravelly sand aquifer. While ERT provided superior vertical resolution and a detailed view of shallow subsurface features, its resolution diminished at greater depths, highlighting the need for deeper exploration. TEM addressed this limitation by offering a detailed conductivity profile at greater depths, detecting layers such as contaminated coal and clay layers, and extending the interpretation of subsurface conditions beyond the depth range of ERT. This integration provided a comprehensive delineation of subsurface features, where each method addressed each other limitations. Magnetic surveys efficiently localized metallic objects and landfill boundaries, while ERT and TEM together offered high-resolution imaging and conductivity profiling, particularly of contamination pathways and geological structures. This integrated geophysical approach not only enhanced site characterization but also revealed complex subsurface interactions that could not have been captured by any single method. The synergy of these techniques demonstrates the value of combining geophysical data to address environmental challenges at landfills, providing a robust and multidimensional analysis of waste composition, contamination migration, and their broader environmental implications.

## Conclusions

The integrated geophysical approach employed at the Weidenpesch landfill effectively combines magnetic, ERT, and TEM methods to provide a comprehensive subsurface characterization. This multi-method approach revealed a waste body extending up to 10–15 m deep with resistivity value range of 1–10 Ω·m in the western and central areas of the landfill and 20–50 Ω·m at the eastern part. Beneath this, a gravelly sand layer was detected at depths of 20–23 m with resistivity values of 80–100 Ω·m. Below that, a highly conductive coal/clay layer, with resistivity values of less than 5 Ω·m, was observed. The geophysical results consistently highlighted lower resistivity values within the gravelly sand layer beneath the landfill and a significant reduction in resistivity in the coal/clay layer compared with their values outside the landfill, suggesting a possible contamination migration. The TEM results further demonstrated increased conductivity in the coal/clay layer over time. The consistency among the methods confirms the robustness of the findings. The results of the study provide important information about the current situation of the landfill, which would help the Environmental Office of the City of Cologne and other local authorities.

Future studies should incorporate additional techniques, such as induced polarization (IP), for enhanced characterization. Long-term time-lapse monitoring using TEM, ERT, and IP techniques, along with continuous borehole measurements of temperature, groundwater levels, and conductivity will be essential for tracking contamination and guiding effective remediation efforts. This integrated approach not only advances waste management practices but also supports more effective environmental protection and sustainable waste management strategies.

## Data Availability

Data sets generated during the current study are available from the corresponding author on reasonable request.

## References

[1] Alao, J. O. et al. Effects of dumpsite leachate plumes on surface and groundwater and the possible public health risks. *Sci. Total Environ.* **897**, 165469. 10.1016/j.scitotenv.2023.165469 (2023). 37442480 10.1016/j.scitotenv.2023.165469

[2] El-Saadony, M. T. et al. Hazardous wastes and management strategies of landfill leachates: A comprehensive review. *Environ. Technol. Innov.* **31**, 103150. 10.1016/j.eti.2023.103150 (2023).

[3] Tezkan, B. A. Review of environmental applications of quasi-stationary electromagnetic techniques. *Surv. Geophys.* **20**, 279–308. 10.1023/A:1006669218545 (1999).

[4] Soupios, P. & Ntarlagiannis, D. Characterization and monitoring of solid waste disposal sites using geophysical methods: Current applications and novel trends. In *Modelling Trends in Solid and Hazardous Waste Management* (eds Sengupta, D. & Agrahari, S.) 75–103 (Springer, 2017). 10.1007/978-981-10-2410-8_5.

[5] Ibraheem, I. M., Tezkan, B. & Bergers, R. Integrated interpretation of magnetic and ERT data to characterize a landfill in the North-West of Cologne, Germany. *Pure Appl. Geophys.* **178**, 2127–2148. 10.1007/s00024-021-02750-x (2021a).

[6] Guinea, A., Bicknell, J., Cox, N., Swan, H. & Simmons, N. Characterization of legacy landfills with electrical resistivity tomography; a comparative study. *J. Appl. Geophys.* **203**, 104716. 10.1016/j.jappgeo.2022.104716 (2022).

[7] Gazoty, A., Fiandaca, G., Pedersen, J., Auken, E. & Christiansen, A. V. Mapping of landfills using time-domain spectral induced polarization data: The Eskelund case study. *Near Surf. Geophys.* **10**, 575–5861. 10.3997/1873-0604.2012046 (2012).

[8] Ibraheem, I., Tezkan, B. & Bergers, R. Imaging of a waste deposit site near Cologne city, Germany using magnetic and ERT methods. In *The 25th European Meeting of Environmental and Engineering Geophysics, Near Surface Geoscience Conference and Exhibition 8–12 September 2019, The Hague, Netherlands* (2019). 10.3997/2214-4609.201902492.

[9] Dagwar, P. P. & Dutta, D. Landfill leachate a potential challenge towards sustainable environmental management. *Sci. Total Environ.* **926**, 171668. 10.1016/j.scitotenv.2024.171668 (2024). 38485011 10.1016/j.scitotenv.2024.171668

[10] Zari, M. Characteristics and impact assessment of municipal solid waste (MSW). In *Technical Landfills and Waste Management* (eds Anouzla, A. & Souabi, S.) 93–113 (Springer Water, Springer, 2024). 10.1007/978-3-031-52633-6_3.

[11] Baawain, M. S., Al-Futaisi, A. M., Ebrahimi, A. & Omidvarborna, H. Characterizing leachate contamination in a landfill site using TDEM (time domain electromagnetic) imaging. *J. Appl. Geophys.* **151**, 73–81. 10.1016/j.jappgeo.2018.02.002 (2018).

[12] Kondracka, M., Stan-Kłeczek, I., Sitek, S. & Ignatiuk, D. Evaluation of geophysical methods for characterizing industrial and municipal waste dumps. *Waste Manag.* **125**, 27–39. 10.1016/j.wasman.2021.02.015 (2021). 33667980 10.1016/j.wasman.2021.02.015

[13] Song, S. Y., Kim, B., Jeong, J., Park, S. & Nam, M. J. 4D interpretation of time-lapse electrical resistivity monitoring data to identify preferential flow path in a landfill, South Korea. *Environ. Monit. Assess.* **195**, 625. 10.1007/s10661-023-11149-1 (2023). 37119389 10.1007/s10661-023-11149-1

[14] De Donno, G., Melegari, D., Paoletti, V. & Piegari, E. Electrical and electromagnetic prospecting for the characterization of municipal waste landfills: A review. In *Technical Landfills and Waste Management* (eds Anouzla, A. & Souabi, S.) (Springer Water, 2024). 10.1007/978-3-031-52633-6_1.

[15] Casado, I., Mahjoub, H., Lovera, R., Fernandez, J. & Casas, A. Use of electrical tomography methods to determinate the extension and main migration routes of uncontrolled landfill leachates in fractured areas. *Sci. Total Environ.* **506**, 546–553. 10.1016/j.scitotenv.2014.11.068 (2015). 25433381 10.1016/j.scitotenv.2014.11.068

[16] Helene, L. P. I., Moreira, C. A. & Bovi, R. C. Identification of leachate infiltration and its flow pathway in landfill by means of electrical resistivity tomography (ERT). *Environ. Monit. Assess.* **192**, 249. 10.1007/s10661-020-8206-5 (2020). 32211981 10.1007/s10661-020-8206-5

[17] Helene, L. P. I. & Moreira, C. A. Analysis of leachate generation dynamics in a closed municipal solid waste landfill by means of geophysical data (DC Resistivity and Self-potential methods). *Pure Appl. Geophys.* **178**, 1355–1367. 10.1007/s00024-021-02700-7 (2021).

[18] Morita, A. K. M., Pelinson, N. S., Bastianon, D., Saraiva, F. A. & Wendland, E. Using electrical resistivity tomography (ERT) to assess the effectiveness of capping in Old Unlined landfills. *Pure Appl. Geophys.* **180**, 3599–3606. 10.1007/s00024-023-03346-3 (2023).

[19] Zaini, M. S. I. & Hasan, M. Application of electrical resistivity tomography in landfill leachate detection assessment. In *A Review of Landfill Leachate* (eds Anouzla, A. & Souabi, S.) 1–22 (Springer Water, Springer, 2024). 10.1007/978-3-031-55513-8_1.

[20] Appiah, I., Wemegah, D. D., Asare, V. D. S., Danuor, S. K. & Forson, E. D. Integrated geophysical characterisation of Sunyani municipal solid waste disposal site using magnetic gradiometry, magnetic susceptibility survey and electrical resistivity tomography. *J. Appl. Geophys.* **153**, 143–153. 10.1016/j.jappgeo.2018.02.007 (2018).

[21] Godio, A. & Chiampo, F. Geophysical monitoring of leachate injection in pretreated waste landfill. *Appl. Sci.* **13**, 5661. 10.3390/app13095661 (2023).

[22] Mary, B. et al. Non-invasive investigations of closed landfills: An example in a karstic area. *Sci. Total Environ.* **905**, 167083. 10.1016/j.scitotenv.2023.167083 (2023). 37730071 10.1016/j.scitotenv.2023.167083

[23] Sun, X. et al. LDI-MVFNet: A multi-view fusion deep network for leachate distribution imaging. *Waste Manag.* **157**, 180–189. 10.1016/j.wasman.2022.12.020 (2023). 36563516 10.1016/j.wasman.2022.12.020

[24] Ibraheem, I. M. Geophysical potential field studies for developmental purposes at El-Nubariya—Wadi El-Natrun area, West Nile Delta, Egypt. Ph.D. thesis, Faculty of Science, Mansoura University, Mansoura 144–160 (2009).

[25] Feng, S. J., Zhao, Y., Zhang, X. L. & Bai, Z. B. Leachate leakage investigation, assessment and engineering countermeasures for tunneling underneath a MSW landfill. *Eng. Geol.* **265**, 105447. 10.1016/j.enggeo.2019.105447 (2020).

[26] Ibraheem, I. M., Othman, A. & Ghazala, H. Pliocene aquifer characterization using TEM and VES geophysical techniques: Case study at the area to the East of Wadi El-Natrun City, West Nile Delta, Egypt. In *Sustainability of Groundwater in the Nile Valley, Egypt. Earth and Environmental Sciences Library* (eds Negm, A. M. & El-Rawy, M.) 235–266 (Springer, 2022). 10.1007/978-3-031-12676-5_10.

[27] Sharifi, F., Tezkan, B., Ibraheem, I. M., Bergers, R. & Yogeshwar, P. Recovering Induced Polarization effects from 1-D coupled inversion of transient Electromagnetic Data. *Geophys. J. Int.* **238** (3), 1708–1722. 10.1093/gji/ggae237 (2024).

[28] Smirnova, M. et al. 3-D radio-frequency CSEM at the Weidenpesch waste site in Cologne. In *The 25th EM Induction Workshop 11–17 September 2022, Çeşme, Turkey* 1–4 (2022).

[29] Fadavi, S. et al. 3D Inversion of Radio-Magnetotelluric (RMT) and Controlled-Source RMT (CSRMT) Data of a Waste-Site in Cologne, Germany. In *The 29th European Meeting of Environmental and Engineering Geophysics, Near Surface Geoscience Conference and Exhibition 03–07 Sep. 2023, Edinburgh, United Kingdom* 1–5 (2023). 10.3997/2214-4609.202320050

[30] Tezkan, B. et al. A joint application of radiomagnetotellurics and transient electromagnetics to the investigation of a waste deposit in Cologne (Germany). *J. Appl. Geophys.* **34**, 199–212. 10.1016/0926-9851(95)00016-X (1996).

[31] Schmitz, K. J. *Gutachten zur Gefährdungsabschätzung der Altablagerung Graseggerstraße, Köln-Longerich (A50502)* (Institut für Umweltgeologie, 1989).

[32] Heuser, H. & Thielmann, G. *Ingenieurgeologische Karten 1:25000/Köln Map* 1st edn. (Geologischer Dienst Nordrhein, 1986).

[33] DABO - Datenbank Aufschlüsse und Bohrungen, Geologischer Dienst NRW; https://www.bohrungen.nrw.de/, license acc. http://www.govdata.de/dl-de/by-2-0, Accessed 13 June 2023.

[34] Schlimm, W. Grundwasser-Dargebot, Nutzung Und Gefährdung. In *Geologie am Niederrhein* (ed Hilden, H. D.) (Geologisches Landesamt Nordrhein-Westfalen, 78–86) (1988).

[35] Kotowski, C. & Fröhlich, D. *Gefährdungsabschätzung Der Altedeponie 50502 Grasegger Straße in Köln-Longerich, Gutachten* (GFM Umwelttechnik, 2007).

[36] Ibraheem, I. M., El-Husseiny, A. A. & Othman, A. A. Structural and mineral exploration study at the transition zone between the North and the Central Eastern Desert, Egypt, using airborne magnetic and gamma-ray spectrometric data. *Geocarto Int.* **37**(26), 13098–13126. 10.1080/10106049.2022.2076915 (2022).

[37] Ibraheem, I. M., Bergers, R. & Tezkan, B. Archaeogeophysical exploration in Neuss-Norf, Germany using electrical resistivity tomography and magnetic data. *Near Surf. Geophys.* **19** (5), 603–623. 10.1002/nsg.12172 (2021).

[38] Loke, M. H. *Tutorial: 2-D and 3-D Electrical Imaging Surveys* (Geotomosoft Solutions, 2024).

[39] Gomaa, M. M., Zarif, F., Shenawy, A. E., Ramah, M. & Kotb, A. D. M. Modelling and simulating the geoelectrical attributes of near-surface buried objects to optimizing its discovery. *Model. Earth Syst. Environ.*10.1007/s40808-024-02095-z (2024).

[40] Dosoky, W. Assessment of three mixed arrays dataset for subsurface cavities detection using resistivity tomography as inferred from numerical modelling. *SN Appl. Sci.* **5**, 303. 10.1007/s42452-023-05539-w (2023).

[41] Loke, M. H., Acworth, I. & Dahlin, T. A comparison of smooth and blocky inversion methods in 2D electrical imaging surveys. *Explor. Geophys.* **34**, 182–187. 10.1071/EG03182 (2003).

[42] Dahlin, T. & Zhou, B. A numerical comparison of 2D resistivity imaging with 10 electrode arrays. *Geophys. Prospect.* **52**, 379–398. 10.1111/j.1365-2478.2004.00423.x (2004).

[43] Nabighian, M. N. & Macnae, J. C. Time domain electromagnetic prospecting methods. In *Electromagnetic Methods in Applied Geophysics-Applications Part A* (ed. Nabighian, M. N.) 427–520 (Society of Exploration Geophysicists, 1991). 10.1190/1.9781560802686.ch6.

[44] Yogeshwar, P. et al. Innovative boat-towed transient electromagnetics—Investigation of the Furnas volcanic lake hydrothermal system, Azores. *Geophysics* **85** (2), E41–E56. 10.1190/geo2019-0292.1 (2020).

[45] Tikhonov, A. N. & Arsenin, V. A. *Solution of Ill-Posed Problems* (Winston & Sons, 1977).

[46] Constable, S. C., Parker, R. L. & Constable, C. G. Occam’s inversion: A practical algorithm for generating smooth models from EM sounding data. *Geophysics* **52** (3), 289–300. 10.1190/1.1442303 (1987).

[47] Marquardt, D. An algorithm for least squares estimation of non-linear parameters. *J. Soc. Ind. Appl. Math.* **11** (2), 431–441. 10.1137/0111030 (1963).

[48] Meju, M. A. Geophysical data analysis: Understanding inverse problem in theory and practice. *Soc. Explor. Geophys.*10.1190/1.9781560802570 (1994).

[49] Yogeshwar, P. & Tezkan, B. Analysing two-dimensional effects in central loop transient electromagnetic sounding data using a semi-synthetic tipper approach. *Geophys. Prospect.* **66** (2), 444–456. 10.1111/1365-2478.12520 (2018).

[50] Ward, S. H. & Hohmann, G. W. Electromagnetic theory for geophysical applications. In *Electromagnetic Methods in Applied Geophysics* Vol. 1 (ed. Nabighian, M. N.) 220–221 (Theory. Society of Exploration Geophysicists, 1988). 10.1190/1.9781560802631.ch4.

[51] Spies, B. R. & Frischknecht, F. C. Electromagnetic sounding. In *Electromagnetic Methods in Applied Geophysics-Applications Part A* (ed. Nabighian, M. N.) 285–425 (Society of Exploration Geophysicists, 1991). 10.1190/1.9781560802686.ch5.

[52] Deutscher Wetterdienst, M. & Klimastatus, Datenteil für September 2021. https://www.dwd.de/DE/leistungen/pbfb_verlag_monat_klimastatus/monat_klimastatus.html (2021), accessed on 25 October 2024.

